# A Global Feature-Rich Network Dataset of Cities and Dashboard for Comprehensive Urban Analyses

**DOI:** 10.1038/s41597-023-02578-1

**Published:** 2023-09-30

**Authors:** Winston Yap, Filip Biljecki

**Affiliations:** 1 Department of Architecture, National University of Singapore, Singapore, Singapore; 2 Department of Real Estate, National University of Singapore, Singapore, Singapore

**Keywords:** Geography, Sustainability

## Abstract

Urban network analytics has become an essential tool for understanding and modeling the intricate complexity of cities. We introduce the Urbanity data repository to nurture this growing research field, offering a comprehensive, open spatial network resource spanning 50 major cities in 29 countries worldwide. Our workflow enhances OpenStreetMap networks with 40 + high-resolution indicators from open global sources such as street view imagery, building morphology, urban population, and points of interest, catering to a diverse range of applications across multiple fields. We extract streetscape semantic features from more than four million street view images using computer vision. The dataset’s strength lies in its thorough processing and validation at every stage, ensuring data quality and consistency through automated and manual checks. Accompanying the dataset is an interactive, web-based dashboard we developed which facilitates data access to even non-technical stakeholders. Urbanity aids various GeoAI and city comparative analyses, underscoring the growing importance of urban network analytics research.

## Background & Summary

Urban networks offer a powerful and intuitive lens to view, understand, and model the complexity of cities^1–6^. Presently, network analytics is employed to optimise decision-making procedures across all urban scales, ranging from coordinating city-wide vehicle fleets to the planning and design of active mobility systems^7,8^. Machine learning and predictive GeoAI offer numerous untapped opportunities to extract valuable insights from urban networks and expand existing use cases^9–12^. While significant progress has been made, the task of generalising machine learning methods to urban networks remains a critical challenge. Specifically, constraints in data consistency and interoperability, model explainability, and the feature representation of varied built environment features within networks make this a complex task^13–15^. Moreover, current graph-based learning methods continue to prejudice a technical interpretation of urban streets based largely on the structural properties of networks, despite emerging evidence that graph algorithms learn from both structural and attribute-based features^16^. Towards advancing analytical and methodological innovation in urban networks, uniform, contextually comprehensive, and open spatial network datasets can serve as an invaluable resource for the urban research community. Good feature representation not only helps to improve model performance but makes it easier for domain experts and decision-makers to understand and interpret the results of GeoAI models. This is particularly important in urban applications where the rationale behind the model’s predictions needs to be transparent and explainable. Developments in related built environment domains have demonstrated the wide-ranging potential of open datasets to unify community analytical efforts and cultivate a more rigorous and critical urban science^17–21^.

Urban streets, serving as multifaceted channels of city life, naturally lend themselves to modeling urban networks. Although various network representations exist due to diverse analytical motivations across urban disciplines, primal planar road networks have emerged as the predominant representation in modern efforts^22–27^. These networks depict road segments as edges and intersections as nodes^28^. Primal planar networks have gained prominence for their geometric fidelity, data and tool interoperability, and use case flexibility, making them particularly useful and effective for a wide range of applications.

Primal planar networks preserve the geometric accuracy of urban streets in 2D euclidean space, resulting in a visually intuitive model that facilitates interpretation and communication among researchers, urban planners, and policymakers. Furthermore, these networks are compatible with numerous geospatial data types, allowing for seamless integration with popular geospatial analytical tools and techniques^29–34^. This compatibility enables primal planar representations to directly benefit from the growing availability of spatially accurate, location-based urban data, including social media check-ins, business location data, and crowdsourced information^35^.

For urban and regional planning, primal planar graphs supplemented with contextual built environment data can support various applications and use cases. These networks are essential in transportation planning for assessing traffic flow, identifying bottlenecks, and optimizing road networks^36–38^. Network population estimates can also help evaluate the accessibility of public facilities, such as schools, hospitals, or parks, empowering planners to pinpoint underserved areas and prioritise infrastructure investments^39–41^. Street view indicators along network edges are crucial for modeling pedestrian-friendly urban environments and suggesting improvements to promote walking, cycling, and other active transportation modes^42–45^ In emergency planning, identifying critical nodes and links can inform strategies to enhance city resilience against various shocks and stresses, including climate change, natural disasters, or economic fluctuations. Lastly, building morphology information along networks plays a vital role in energy-based modeling and carbon forecasting for urban areas, providing insights into the implications of urban growth for social, economic, and environmental outcomes^19,46^.

The growing availability of open urban data presents opportunities to construct a global-scale network dataset of cities with rich contextual and semantic embeddings, including street view imagery, building morphology, points of interest, and urban population indicators. However, to our knowledge, no such dataset currently exists for individual cities or urban regions. Existing projects, such as the Stanford Network Analysis Project (SNAP) and the Network Data Repository, offer undirected road networks for investigating structural network attributes, but feature representation is limited to structural and topological indicators^47,48^. Similarly, the Global Urban Street Networks project offers a comprehensive repository that encompasses both directed and undirected geometric and topological properties of urban networks^49^. Currently, users also experience considerable entry barriers since substantial software expertise is required to effectively visualise and analyze network data. Although the OpenStreetMap (OSM) project hosts a comprehensive crowdsourced collection of road networks and points of interest, raw OSM network data often suffer from data consistency issues and lack useful network indicators^32,50,51^.

This paper introduces the Urbanity dataset^52^, which spans 50 global cities across 29 countries, overcoming these limitations. Urbanity^53^ collects comprehensive spatial information on urban network elements, supporting various urban applications and use cases in urban planning and research. Our open and consistent workflow ensures reproducibility of urban networks and enables comparative analyses between cities. We ensure high usability and consistency of generated urban networks through meticulous data screening, pre-processing, and harmonisation efforts. Extensive data validation, involving both automated and manual checks, is performed throughout the process to guarantee data quality and consistency. Our work expands upon previous efforts in several ways: (1) we develop a completely open workflow to generate urban networks; (2) we create and augment city networks with rich contextual and semantic indicators; (3) we build an interactive visual dashboard that makes urban network data accessible even to non-technical users.

The dataset is available under a Creative Commons Attribution 4.0 International (CC BY 4.0) license and it is hosted on Figshare (10.6084/m9.figshare.22124219)^52^. All source code used to generate and validate the dataset are available under an open-source MIT license (https://github.com/winstonyym/urbanity). The Urbanity dashboard source code is fully accessible (https://github.com/winstonyym/urbdash).

## Methods

**Fig. 1:**
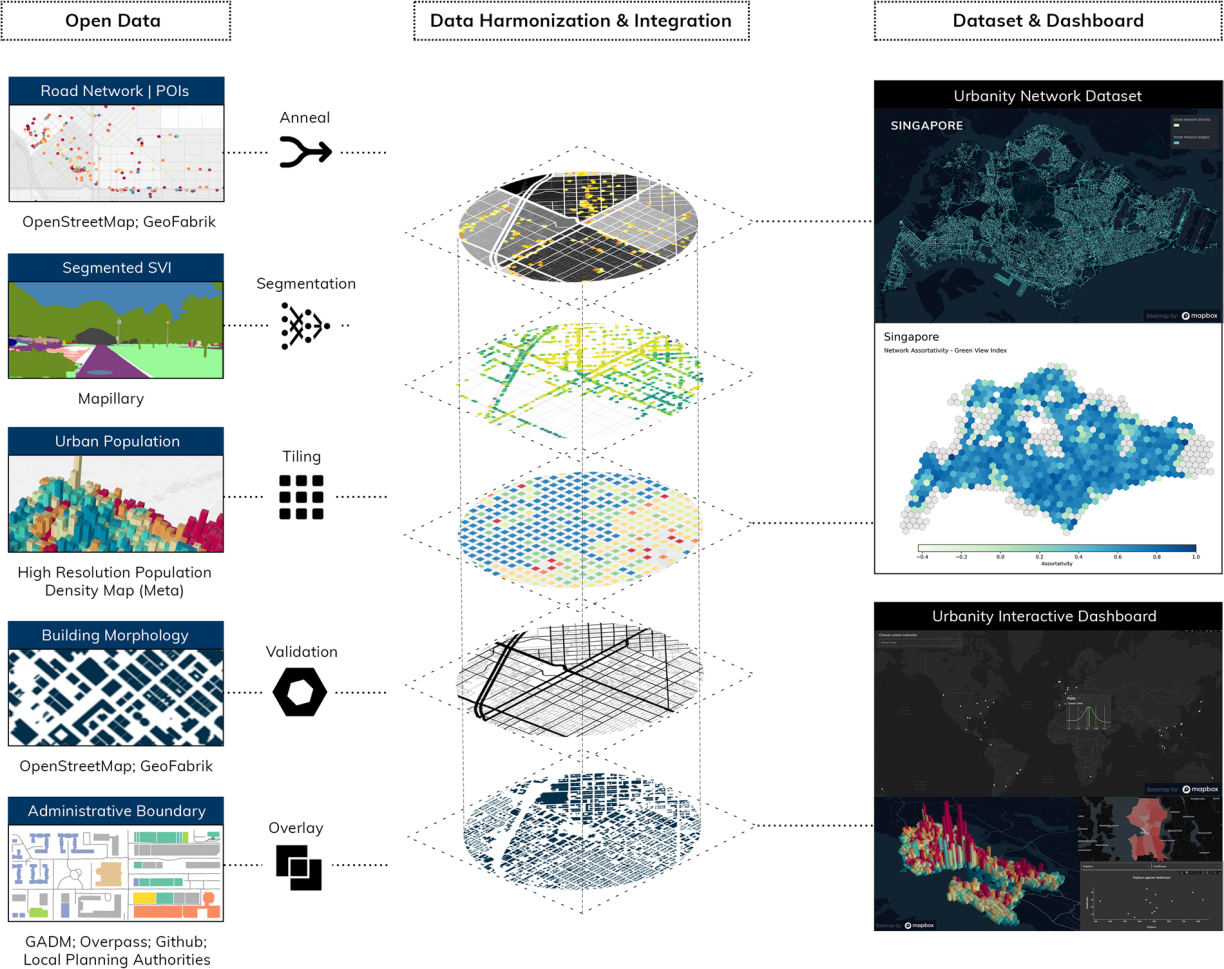
Overview of open workflow. Our workflow integrates urban data from heterogeneous open layers and provides a consistent framework to construct feature rich urban networks of global cities. Sources of the data samples: (c) OpenStreetMap contributors, Mapillary, Meta. Basemap: OpenStreetMap and Mapbox.

Our data workflow consists of the following three main steps: (1) data identification, retrieval, and pre-processing; (2) data harmonisation, generation, and integration into urban networks; (3) dashboard conceptualisation and development. We employ a consistent and standardised analytical pipeline to pre-process open data from various built environment domains (population, street view imagery, building morphology, and urban amenities). Figure 1 provides an overview of our workflow.

### Data identification, retrieval, and pre-processing

Several considerations factor into the selection of urban datasets: (1) open source; (2) use case suitability. A key aim of our data set is to promote open benchmarking and comparative analyses of global cities. Open benchmarking and comparative analysis help to promote urban innovation, optimise resource allocation, and facilitate the transfer of knowledge between cities. For this purpose, we selected open datasets with reasonable global coverage. Another motivation for data selection is to continue supporting the open source eco-system. Free and open source projects have been one of the main contributors of planning technologies innovation in the last decade^54^. Recent breakthrough technologies such as the ChatGPT series were almost entirely developed on open source technologies and information^55^. For this reason, we selected datasets with open access licenses to faciliate usage for downstream analytical purposes. Another important consideration is use case suitability. We see an increasing trend among urban network analytical use cases to use spatially granular location data for local scale prediction. For such cases, coarse urban environment data could negatively impact model predictive performance by masking fine-grained heterogeneity along urban networks. To address this concern, we made the decision to include population data with high spatial resolution. These conditions imply that some popular datasets such as Google Street View, WorldPop, and the Global Human Settlement Layer were omitted due to either proprietary or granularity reasons.

Data retrieval is a non-trivial task given the size, scope, and diversity of data. To create a consistent and standardised data collection process, we developed customised workflows to automate data extraction and pre-processing.

### Population data

High resolution population data is essential to support planning efforts, playing a critical role in infrastructure planning and resource distribution. We obtain population data from Meta’s high-resolution (30-metre) population density maps which is built from global satellite imagery and census data^56,57^. Population data is updated on an annual basis by Meta and currently unavailable for Russia, Ukraine, and Mainland China. The data set is released under a Creative Commons Attribution International (CC BY 4.0) which permits users to freely share and adapt the data set. Each country’s population file is distributed as either Tag Image File Format (TIFF) or Comma Separated Values (CSV) and includes population counts for socio-demographic groups such as total population, women, men, elderly, youths, and children. For large geographic areas (e.g., United States), population data is further split into multiple files. Meta provides two options for information retrieval: (1) Humanitarian Data Exchange (HDX) or (2) Amazon Web Services (AWS). Since HDX is free and allows for direct download for desktop pre-processing we opt for the HDX approach (metadata and data files available at: https://dataforgood.facebook.com/dfg/tools/high-resolution-population-density-maps). Accordingly, we webscrape uniform resource locator (URL) from HDX for all countries, socio-demographic groups, and file types. The most recent population statistics are now accessible for the year 2020. In the course of our analysis, we noticed that the data files for certain countries include supplementary columns related to the preceding year, 2019. To ensure uniformity across all cities, even those without 2019 data, our dataset exclusively reports population figures for the year 2020.

### Street network, points of interest, and building footprints

We extract street networks, points of interest, and building footprint data from OpenStreetMap (OSM). OSM is an open collaborative mapping platform that hosts the most comprehensive global crowdsourced collection of geospatial data^58^. The data from OpenStreetMap (OSM) is released under the Open Data Commons Open Database License (ODbL). Access to OSM data is facilitated via the Pyrosm API, granting users entry to raw, regularly updated OSM data sourced from GeoFabrik. This method serves to avert potential bottlenecks that could arise from repeated queries to OSM’s Overpass API.

**Fig. 2:**
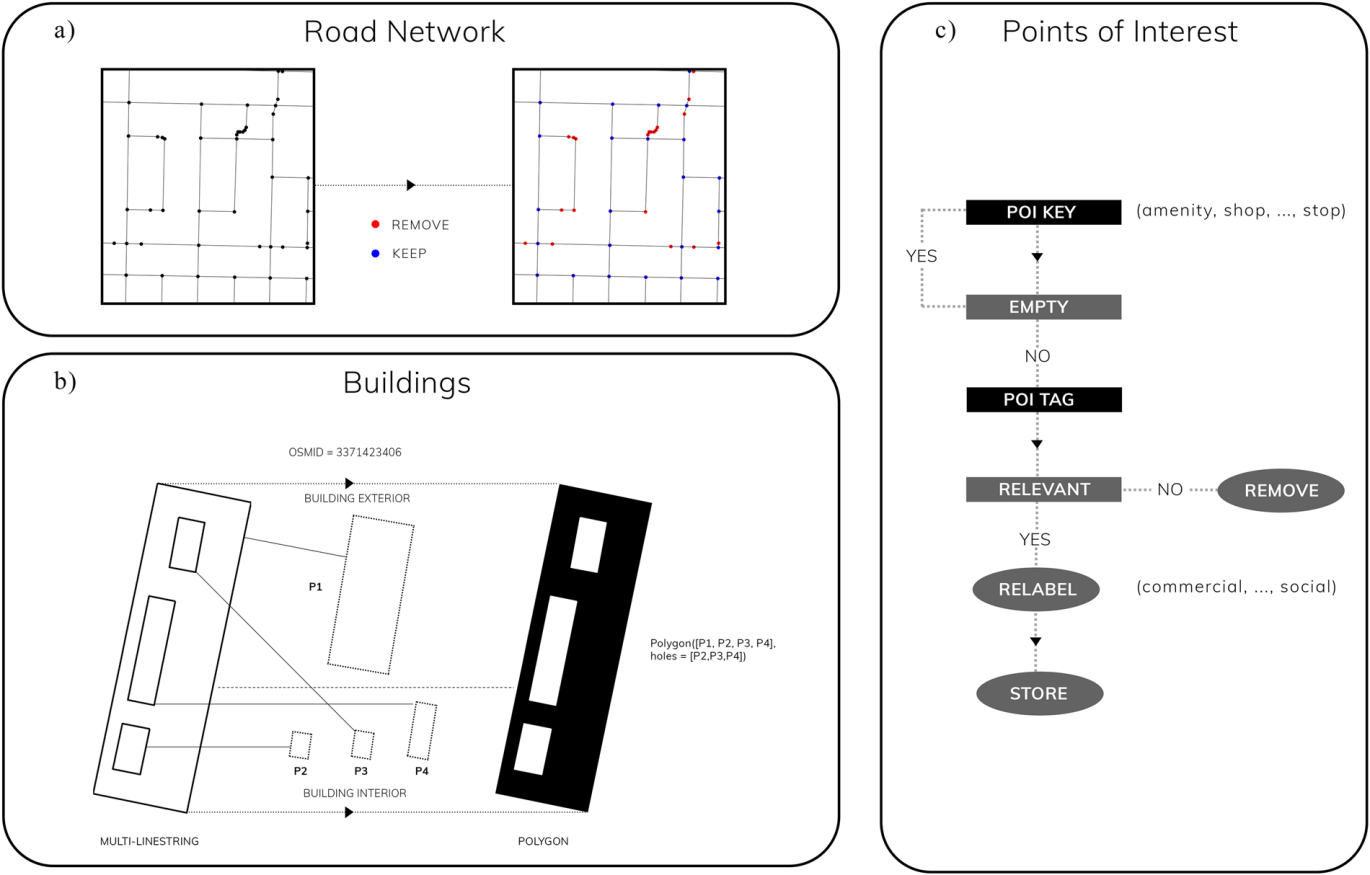
OpenStreetMap preprocessing workflow. Flowchart of OpenStreetMap preprocessing workflow for road networks, building footprints, and urban points of interests. Sources of the data samples: (c) OpenStreetMap contributors. (**a**) Road networks are topologically simplified to primal planar representation. (**b**) Building footprints are converted to valid polygons. (**c**) Points of interest are retrieved, procedurally checked, and re-labelled.

Figure 2 presents an overview of our pre-processing workflow for OSM data. For street networks, we pre-process raw road networks by removing nodes that do not conform to primal planarity^32^. This helps to simplify network structure and reduce over-estimation of node degree in networks^22^. Points of Interest (POI) entries are recorded under OSM’s primary key tags (amenity, shop, tourist, leisure). However, not all tags correpond directly to urban amenities. To address this issue, we manually inspect and choose relevant tags under each primary key tag. For example, we extract ‘museum’, ‘gallery’, ‘artwork’, and ‘attraction’ from the tourist primary key tag. A further pre-processing step was employed to deal with duplicate tagging. For instance, some amenities were found to be tagged with multiple labels (e.g., amenity and shop). To prevent double counting, we apply procedural selection across each observation. More specifically, we first check if the amenity field is empty, and if it is, we check for values in the order of tourist, leisure, and shop. Finally, we relabel the list of amenities according to eight main categories–civic, recreational, entertainment, food, healthcare, institutional, social, and commercial. While we strive to establish useful urban categories, we acknowledge that any approach to urban classification remains a complex and subjective endeavour due to the diverse and eclectic nature of human systems which span many cultures and disciplines. In line with this viewpoint, our software grants users the adaptability to harmonise POI categorisations with their precise needs and the distinct local contexts they are operating within.

For buildings footprints, we implement a procedural script to ensure that all buildings correspond to valid polygons. This step is necessary to ensure that building footprint area can be computed downstream. In particular, we first check the geometric type of each building row and convert line objects into polygons. For objects with multiple lines (e.g., compounds with inner courtyards), we build polygons in a two-step process: (1) identify the exterior building perimeter by geographic extent; (2) build polygon with building perimeter as bounds and interior lines as open space within each building.

### Street view imagery

Street view imagery (SVI) provides a scalable and accessible option for planners to understand the physical characteristics of streetscapes, such as greenery, building cover, and visual complexity. We obtain SVI from Mapillary which is the world’s largest platform for free and open street view imagery. Till date, Mapillary’s coverage has penetrated most global cities around the world thanks to myriads of contributors. Compared to proprietary options such as Google Street View, Mapillary images are hosted under a CC-BY-SA 4.0 licence, which allows users to freely share, use, and adapt images. Images may also be updated more frequently and enjoy better coverage for dense urban areas^44,59^. The latest access point is provided via Mapillary’s Version 4.0 application programming interface (API). To obtain a list of target images that correspond to each city’s boundary, we adopt a two step approach: (1) spatial query intersecting vector tiles (see Mapbox documentation: https://docs.mapbox.com/help/glossary/zoom-level/) for image meta data; (2) spatially filter image points that lie within administrative boundary. Subsequently, we extract meta information such as geographic coordinates, image ID, camera bearing, and timing of image capture. The image segmentation process involves the automated identification and extraction of semantic label masks that represent various visual elements, such as buildings, roads, cars, and greenery. On hardware, we segment SVI images with a NVIDIA Geforce RTX 3090 GPU which allows processing up to 90,000 images daily. Pre-computation enables users to compute SVI indicators within seconds, even if they lack GPU access or expertise in SVI workflows, considerably facilitating such analyses and lowering entry barriers in this domain.

### Data harmonisation, generation, and integration

#### Administrative boundaries

We employ pre-post processing to harmonise spatial data across a variety of data representations and urban scales. First, we manually inspect and extract city administrative boundary and subzone data from various sources–Database of Global Administrative Areas (GADM)(https://gadm.org/), OSM Overpass (https://overpass-turbo.eu/), and local government sites (Germany: https://github.com/codeforgermany/click_that_hood/tree/main/public/data; New Zealand: https://catalogue.data.govt.nz/dataset/auckland-council-boundary-area).

In general, GADM data is provided under the GADM license which permits free usage for academic and non-commercial application (except files for Austria which are shared under the Creative Commons Attribution-ShareAlike 2.0 license). OSM data are provided under the ODbL license and Code for Germany files are provided under an open MIT license. Similarly, files provided by the New Zealand government are released under the CC BY 4.0 license.

OpenAI’s ChatGPT 4 model was helpful in providing OSM Overpass queries for municipal administrative boundaries (e.g., Kowloon and Zagreb). In many cases, administrative boundaries had to be integrated across various sources. For instance, certain cities only had subzone level information for the country level (e.g. GADM Level 0) or wider metropolitan region (GADM Level (1) but not directly for the city level. To deal with such cases, we adopt a general four step spatial harmonisation process: (1) project all shapefiles to common global coordinate reference systems (CRS); (2) obtain spatial entities that spatially intersect city boundary via spatial overlay; (3) filter out spatial entities that do not correspond to valid polygons; (4) visually check spatial correspondence and manually relabel missing subzone names. To the best of our knowledge, there is no existing method capable of automating the manual relabelling process on a large scale. Although we note that the emergence of expansive geospatial foundational models could provide a promising way forward in this domain. We subsequently project administrative boundaries to local CRS and implement a spatial buffer (to account for edge entities) before extracting road network, building, and POIs from GeoFabrik.

#### Urban population

Meta provides population data in both Tag Image File Format (TIFF) and Comma Separated Values (CSV). For some countries, population data is available in only one file format for certain subgroups. To facilitate downstream analytical tasks, we implement workflows to process both raster and tabular formats into a common vector point representation. Raster formats are transformed using an affine transformation matrix (via the Rasterio package) to obtain coordinate representations.

One concern is the poor match between city administrative boundaries and the geographic extent of population data. For example, the population data file for Spain extends beyond its national boundaries, encompassing a large part of North Africa and the Mediterranean Sea. This issue poses significant usability challenges, particularly when users are only interested in population figures at the neighborhood or precinct scale. To address this challenge, we conduct extensive geospatial processing in two main steps: (1) geospatial tiling; and (2) providing an updated geospatial query interface. First, we split larger administrative boundaries into uniform, fixed-size spatial grids (e.g., the entire United States is split into 130 equally sized grid cells). Next, we compute the spatial intersection between population data and their respective grids. Traditional spatial querying with Python geospatial libraries is challenging due to the massive data size (>100 million data points). To address this challenge, we employ the RapidsAI cuSpatial library to implement GPU-accelerated spatial query. As a technical caveat, users seeking to re-implement this approach should set up a Compute Unified Device Architecture (CUDA) enabled Linux local distribution, as cuSpatial is not supported on MacOS or Windows systems. Finally, we merge population data with their respective grids. We release the tiled population dataset and polygon shapefiles for 28 countries (except Singapore) under a CC BY 4.0 license at Figshare (10.6084/m9.figshare.22580806)^60^.

#### Street view imagery

To harmonise semantic classes across diverse urban contexts, we adopt a unified image segmentation pipeline. More specifically, we utilise the ‘Mask2Former’ approach by^61^, a universal transformer architecture applicable to a wide range of image segmentation tasks. Mask2Former is trained and validated on the Mapillary Version 1.2 validation dataset^17^, comprising 65 semantic classes, and reports state-of-the-art performance (MIoU = 63.2%). Mask2Former offers two main advantages for our purposes: (1) improved accuracy to pick out fine-grained semantic categories in images (previous models commonly ignore small regions in images); and (2) lightweight and scalable computation. Readers interested in the specifics of Mask2Former architecture and training are referred to^61,62^. To ensure consistency of daylight conditions for images taken in different cities, we implement timestamp alignment by converting Unix epoch time (POSIX) to local timezones and selecting images taken between 9 am and 5 pm.

#### Node-level integration

This section discusses steps taken to integrate and embed contextual and semantic information into network nodes. A variety of spatial measures have been used to delimit catchment areas and measure access coverage for urban locations. Popular methods include uniform euclidean^63,64^, network-based distance^65^, network voronois^66^, and spatial modelling approaches^9,39^. For node attributes, we adopt a uniform euclidean approach, as it provides a consistent, straightforward, and extensible basis for integrating heterogeneous data sources across different network locations. Accordingly, we construct 100-metre euclidean buffers for each network node and compute the spatial intersection with spatial targets (e.g., street view imagery points, points of interest, and building footprints). In this situation, closely situated nodes might have overlapping spatial areas, which aligns with the concept of urban catchment areas. This recognition reflects the shared geographical context between neighboring nodes. To ensure spatial consistency and accurate distance computation, we project spatial entities into local coordinate reference systems (CRS). To support other use cases, we provide an open source python package where users can generate euclidean buffers of arbitrary distance. As an example, building footprint proportion corresponds to the ratio between the building area and the buffered area around each node^19^.

#### Edge-level integration

To obtain indicators for network edges, we spatially interpolate spatial entities to their nearest network edge^29,65^. More specifically, we adopt a two-step approach: (1) compute the distance between each spatial point of interest and its proximate edges in the network, and (2) assign entities to the corresponding edge with lowest distance. This makes intuitive sense as streets can be characterised by their adjacent amenities. To account for remote edges (e.g., peripheral routes that are not located close to any amenities), we specify a distance threshold of 50 metres. From a computational standpoint, a 50-metre radius effectively encompasses nearby points of interest along edges due to their elongated nature. When we consider the urban context, it makes intuitive sense to use a smaller distance threshold for edges, as edges are meant to encompass elements that are immediately adjacent. For example, a significant portion of buildings are directly adjacent to an edge. For buildings, we compute the distance between building centroids and their respective network edge. Accordingly, we compute spatial indicators based on the set of elements assigned to each network edge.

#### Dashboard conceptualisation and development

**Fig. 3:**
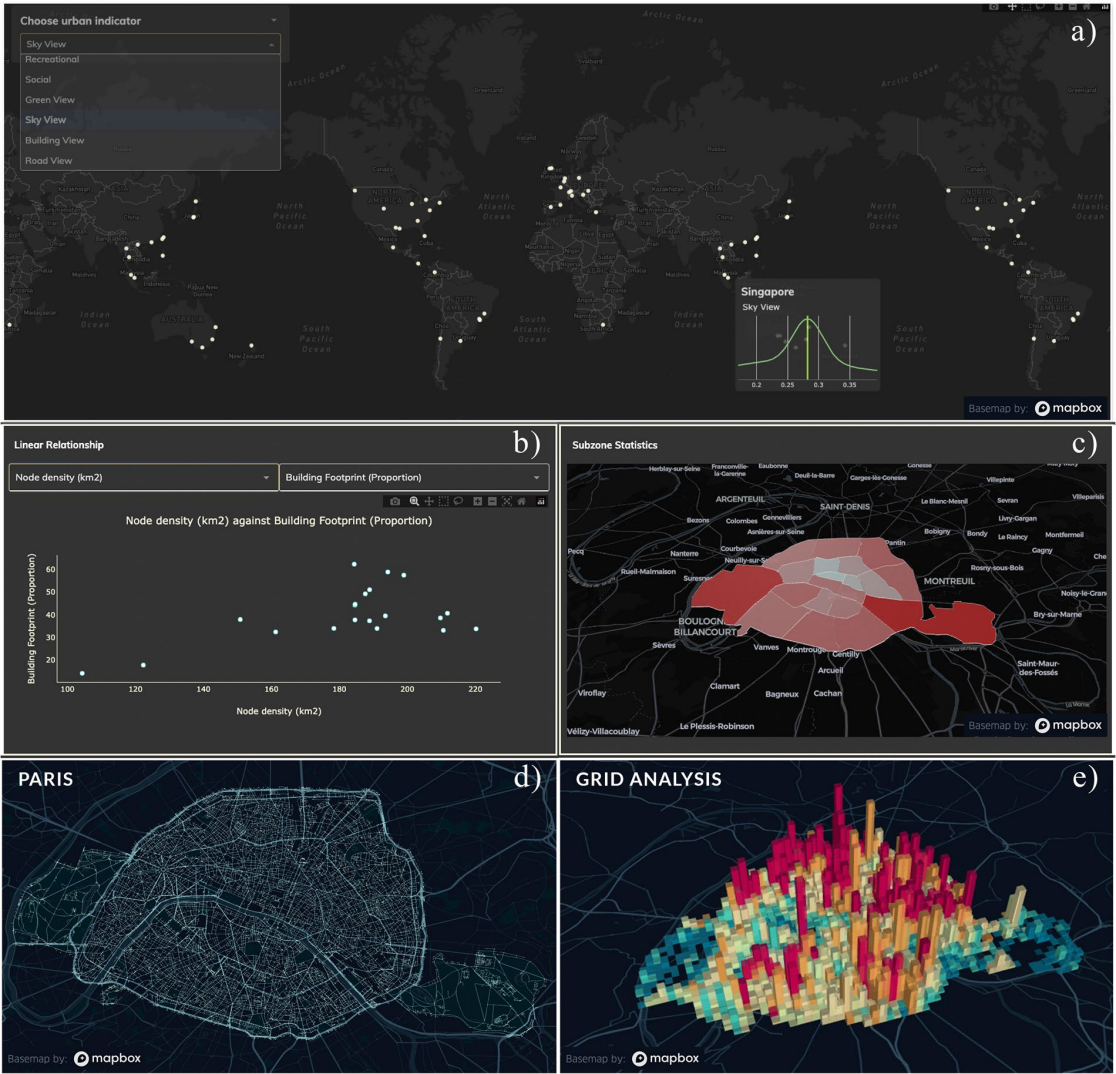
Urbanity dashboard exploratory panels. Urbanity dashboard offers an interactive, user-friendly interface for exploring urban network data without the need to code. Complementary panels provide insights into various urban scales and features, incorporating popular methods for urban network data analysis. Sources of the data samples: (c) OpenStreetMap contributors, Mapillary, and Meta. Basemap: OpenStreetMap and Mapbox. (**a**) Global comparative analyses of cites across selected indicators. (**b**) Bivariate scatterplot of network indicators across city subzones. (**c**) Univariate spatial distribution of aggregate values across city subzones. (**d**) Urban network of Paris. (**e**) Gridded density plot of urban population distribution across Paris.

The Urbanity network dataset^52^ is accompanied by an interactive, web-based dashboard to support comparative analyses and visualisation of network metrics (see Figure 3). Users can use the Urbanity dashboard to examine and compare urban networks through multiple scales–global, city, and local. At the global scale, users can access a variety of network indicators’ distributions (such as building footprint proportions and green view index) across cities worldwide. This functionality helps cities identify their strengths and weaknesses, offering guidance for improvement. Expanding on the global overview, our dashboard provides features to analyze and compare network structures at the city subzone level. As an example of equitable planning, planners can pinpoint infrastructure gaps by evaluating population density and civic facility availability across different subzones. Finally, users can delve into the local scale by directly accessing attributes of nodes and edges. A potential use case would be multi-criteria site assessment which can help planners to quickly identify sites with various characteristics (e.g., low building footprint but high visual complexity). Urbanity dashboard source code is available through an open MIT license (https://github.com/winstonyym/urbdash).

## Data Records

The Urbanity dataset^52^ consists of urban network data for 50 cities across seven regions–Europe (14); Asia (12); North America (11); South America (7); Oceania (5); South Africa (1). We adopt a consistent data workflow to create each city’s network data through the Urbanity Python package (https://github.com/winstonyym/urbanity). Each city’s network consists of two separate Geographic Javascript Object Notation (GeoJSON) files which correspond to attributes and geometry for nodes and edges. Direct spatial representation of nodes and edges allows for rapid visualisation of primal planar networks while also allowing for seamless conversion to popular network formats (NetworkX or igraph). Nodes and edges are assigned unique IDs and retain their original OSM labels. A complete list of node- and edge-level spatial indicators is provided in Table 1.

**Table 1:** List of computed network spatial indicators. ^1^Node features derived from 100-metre euclidean buffers and spatial feature aggregation. ^2^Edge features derived via linear interpolation and assignment of nearby spatial entities to proximate edges. ^3^Recent articles that illustrate the empirical importance of the associated indicator for various urban analytics use cases. ^4^To mitigate the constraints posed by SVI data limitations, we employ a methodology of imputing estimates derived from geographically adjacent neighbors.

Indicator	Node^1^	Edge^2^	Data Type	Unit	References^3^
**Metric & Topological**					
Node Density	Yes		Integer	Count	Boeing^32^ and Huang *et al*.^86^
Street Length	Yes	Yes	Decimal	m	Xue *et al*.^3^
Degree	Yes		Integer	Count	Prieto-Curiel *et al*.^87^
Clustering Coefficient	Yes		Decimal	—	Boeing^49^
Clustering Coefficient (Weighted)	Yes		Decimal	—	Boeing^49^
Closeness Centrality	Yes		Decimal	—	Ozuduru *et al*.^88^
Betweenness Centrality	Yes		Decimal	—	Kirkley *et al*.^89^
Eigenvector Centrality	Yes		Decimal	—	Agryzkov *et al*.^13^
Katz Centrality	Yes		Decimal	—	Curado *et al*.^90^
PageRank	Yes		Decimal	—	Jia *et al*.^91^
**Building Morphology**					
Footprint Proportion (Total)	Yes	Yes	Percentage	m^2^	Asadi *et al*.^92^
Mean Area	Yes	Yes	Decimal	m^2^	Hu *et al*.^93^
Area St. dev	Yes	Yes	Decimal	m^2^	Li *et al*.^94^
Total Perimeter	Yes	Yes	Decimal	m^2^	Tikhonova & Beirao^95^
Mean Perimeter	Yes	Yes	Decimal	m^2^	Litardo *et al*.^96^
Perimeter St. dev	Yes	Yes	Decimal	m^2^	Biljecki & Chow^19^
Complexity Mean	Yes	Yes	Decimal	m^2^	Basaraner & Cetinkaya^97^
Complexity St. dev	Yes	Yes	Decimal	m^2^	Labetski *et al*.^98^
No. of Buildings	Yes	Yes	Integer	Count	Liu *et al*.^99^
**Population**					
Total Population	Yes	Yes	Integer	Count	Szarka & Biljecki^100^
Women	Yes	Yes	Integer	Count	Cerin *et al*.^101^
Men	Yes	Yes	Integer	Count	Gauvin *et al*.^102^
Elderly (aged 60 + )	Yes	Yes	Integer	Count	Wang *et al*.^103^
Youth (15–24)	Yes	Yes	Integer	Count	Ha & Thill^104^
Children (under 5)	Yes	Yes	Integer	Count	Kruse *et al*.^105^
**Points of Interest**					
Social Amenities	Yes	Yes	Integer	Count	Lucchini *et al*.^106^
Recreational Amenities	Yes	Yes	Integer	Count	Klinkhardt *et al*.^107^
Healthcare Amenities	Yes	Yes	Integer	Count	Weiss *et al*.^108^
Entertainment Amenities	Yes	Yes	Integer	Count	Liu *et al*.^109^
Civic Amenities	Yes	Yes	Integer	Count	Liu & Long^110^
Institutional Amenities	Yes	Yes	Integer	Count	Zhou & Yang^111^
Food Amenities	Yes	Yes	Integer	Count	Liu *et al*.^112^
Commercial Amenities	Yes	Yes	Integer	Count	Wang *et al*.^113^
No. of Street View Images		Yes	Integer	Count	Hou & Biljecki^59^
Green View Index	Yes	Yes	Decimal	—	Li^114^
Sky View Index	Yes	Yes	Decimal	—	Middel *et al*.^115^
Building View Index	Yes	Yes	Decimal	—	Ki & Lee^116^
Road View Index	Yes	Yes	Decimal	—	Dong *et al*.^117^
Visual Complexity Index	Yes	Yes	Decimal	—	Yap *et al*.^44^

Indicators were selected and computed according to their perceived empirical importance in urban analytics literature. The dataset is hosted under a CC BY 4.0 license at Figshare (10.6084/m9.figshare.22124219)^52^.

## Technical Validation

In order to conduct meaningful comparative analyses of cities worldwide, the Urbanity dataset^52^ employs data components that feature consistent global jurisdictional coverage. Numerous studies have previously examined the validity and robustness of urban open data. Generally, the OSM community validates OSM data, as described in their documentation (http://wiki.openstreetmap.org/wiki/Accuracy). Significant efforts have been made to evaluate the availability and quality of OSM data in areas such as road networks^51,67–70^, points of interest^71^, and building footprints^58,72–74^. Likewise, several studies have assessed the quality and coverage of street view imagery on crowdsourced platforms like Mapillary and KartaView^59,75,76^. The spatial accuracy of high-density population maps has been systematically validated against population census data in a methodology paper^57^.

Notwithstanding, we employ a multi-level framework involving both automated and manual tests to improve the consistency and reliability of network data components. This framework comprises visual cross-validation, null value and outlier checking, systematic comparisons with available census data, and automated image suitability evaluations, as detailed below.

### Automatic population validation

In this section, we undertake a rigorous assessment of the Meta population dataset through a comprehensive process, comparing it against the well-established WorldPop urban population dataset^77^. Over recent years, WorldPop has gained prominence as a leading open dataset, widely utilised across numerous domains in urban research and decision-making–population health^78,79^, sustainable development^80,81^, socioeconomics^82^. We opted to utilise the WorldPop dataset over comparable alternatives like the Gridded Population of the World (GPW), Global Rural Urban Mapping Project (GRUMP), Global Human Settlement Layer-Population (GHS-POP), or LandScan Population database because of spatial resolution and temporal frequency compatibility between the WorldPop dataset and the Meta population dataset.

**Fig. 4:**
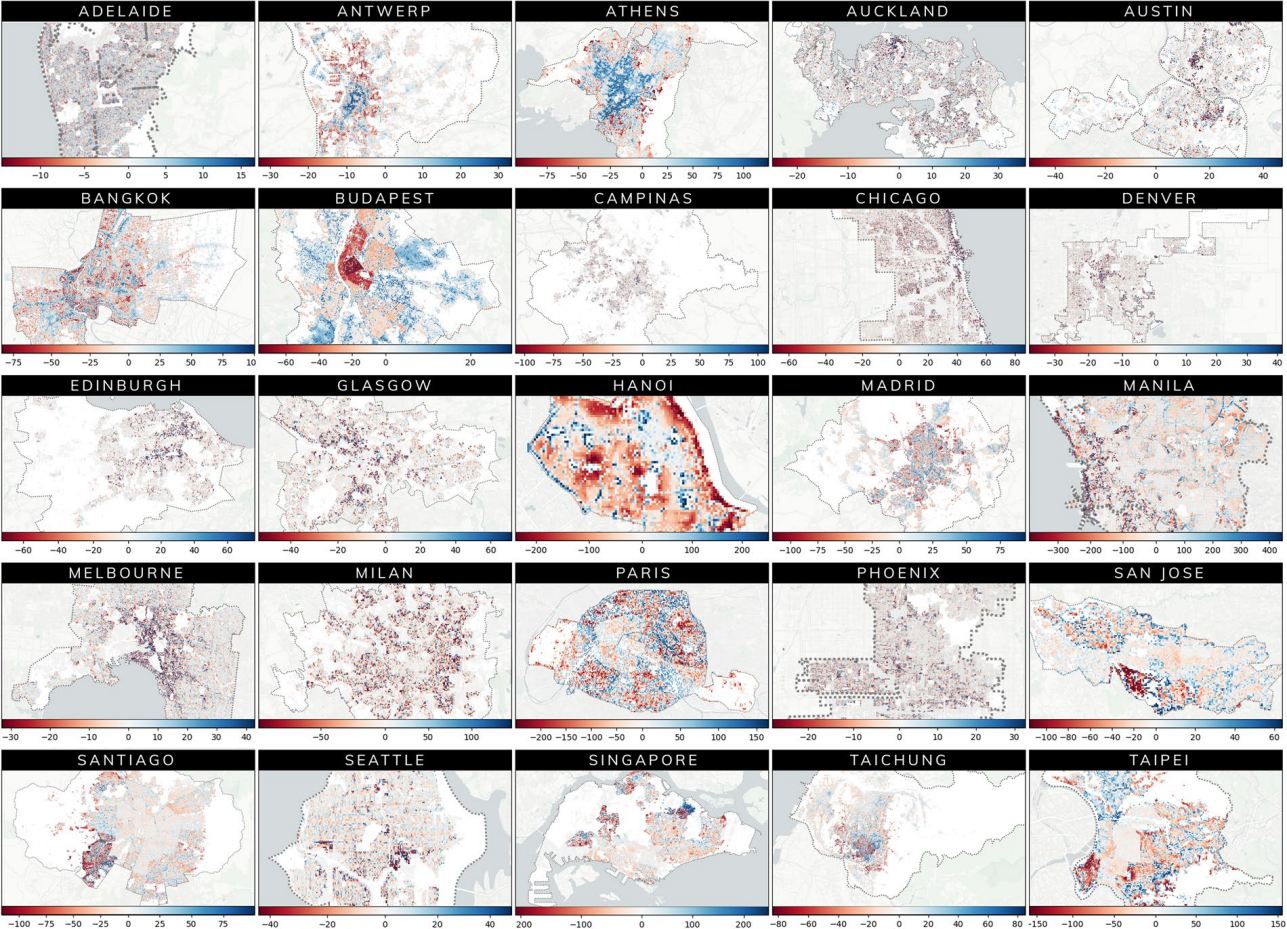
Comparing WorldPop and Meta population datasets using a spatial 100-metre gridded analysis across 25 cities. Highlighting variations in aggregated population counts across administrative boundaries in 25 cities between the WorldPop and Meta Datasets. In the heatmap, regions indicating a higher population count in the Meta dataset compared to WorldPop are visualized in red, while areas where WorldPop predicts a greater population count than the Meta dataset are represented in blue. Data Source: Meta and WorldPop. Basemap: (c) CARTO.

Accordingly, we conduct a systematic comparative analysis of gridded population counts at the 100-metre resolution between the Meta and WorldPop datasets. For the WorldPop dataset, we utilise a top-down approach which employs building footprint constrained and United Nations national population-adjusted figures to disaggregate the population into land cell grids (https://hub.worldpop.org/geodata/listing?id=79). Our analysis encompasses 25 cities from our original dataset, representing diverse geographical regions. For each city, Table 2 enumerates statistics such as mean absolute error, correlation, aggregate population proportion, and the percentage of binary correspondence (zero population/population above zero) between the WorldPop and Meta population datasets. Similarly, Figure 4 displays the spatial distribution of mean absolute error across 100-metre grid cells covering each city.

**Table 2:** Comparison of Meta and WorldPop population datasets across 100-metre grid cells. Cities sorted alphabetically. ^1^MAE—Mean Absolute Error. Corresopnds to the population count difference between the WorldPop and Meta population datasets across 100-metre grid cells for each city’s administrative boundary.

City	Binary Correspondence (Proportion)	Meta Population	WorldPop Population	Correlation	MAE^1^
**Adelaide**	0.754	1,279,450	1,334,155	0.785	4.293
**Antwerp**	0.711	1,158,770	1,057,246	0.543	6.738
**Athens**	0.859	2,508,459	2,935,090	0.669	22.936
**Auckland**	0.865	1,059,257	1,151,367	0.763	7.54
**Austin**	0.785	713,240	715,235	0.758	8.295
**Bangkok**	0.796	13,993,114	14,342,154	0.756	24.816
**Budapest**	0.914	2,002,245	1,826,055	0.54	12.141
**Campinas**	0.66	3,764,216	3,675,747	0.719	17.464
**Chicago**	0.926	3,444,824	3,402,691	0.772	10.008
**Denver**	0.893	1,641,215	1,646,064	0.775	6.531
**Edinburgh**	0.836	507,829	495,732	0.68	12.467
**Glasgow**	0.915	809,246	815,467	0.717	9.95
**Hanoi**	0.958	1,785,191	1,578,247	0.694	73.577
**Madrid**	0.837	5,678,730	5,023,775	0.666	24.01
**Manila**	0.951	15,730,416	15,324,991	0.788	84.387
**Melbourne**	0.982	1,905,490	1,967,284	0.758	6.736
**Milan**	0.901	1,500,331	1,498,404	0.834	18.84
**Paris**	0.984	3,258,001	2,922,765	0.519	61.07
**Phoenix**	0.884	2,450,696	2,444,093	0.779	5.671
**San Jose**	0.944	745,321	695,970	0.706	19.425
**Santiago**	0.886	6,807,438	6,351,675	0.805	25.765
**Seattle**	0.916	698,244	687,313	0.768	7.845
**Singapore**	0.813	5,525,543	5,341,286	0.855	46.614
**Taichung**	0.692	3,217,240	3,137,196	0.824	16.441
**Taipei**	0.796	4,469,983	4,382,751	0.787	54.235

In general, although there are subtle distinctions, our empirical findings underscore a robust and consistent convergence between the two datasets across a range of comparative metrics. Several noteworthy metrics warrant careful consideration. For instance, the significant level of binary correspondence provides compelling evidence that both datasets adeptly forecast the presence or absence of built-up areas within administrative city boundaries. Moreover, when we aggregate population estimates across urban boundaries, the results demonstrate remarkable similarity.

Although there is generally a strong correlation at the grid cell level, it’s important to acknowledge the presence of some variability. This divergence can be attributed to the distinct methodologies employed in disaggregating population data. In the case of the Meta dataset, population counts are directly attributed to building footprints. Conversely, the WorldPop dataset employs a two-step process for population assignment: first, building footprints are predicted using a settlement growth model for buildings; subsequently, population is allocated to grid cells identified as housing buildings or established settlements^83^.

### Automatic SVI assessment

Crowdsourced SVI imagery relies on the contributions of numerous volunteers, which can result in significant variations in image quality^59^. Consequently, it is crucial to evaluate the suitability of these images, as they could adversely affect segmentation results if left unaddressed. We assess the images based on three criteria: (1) perspective; (2) daylight visibility; and (3) occlusion.

**Fig. 5:**
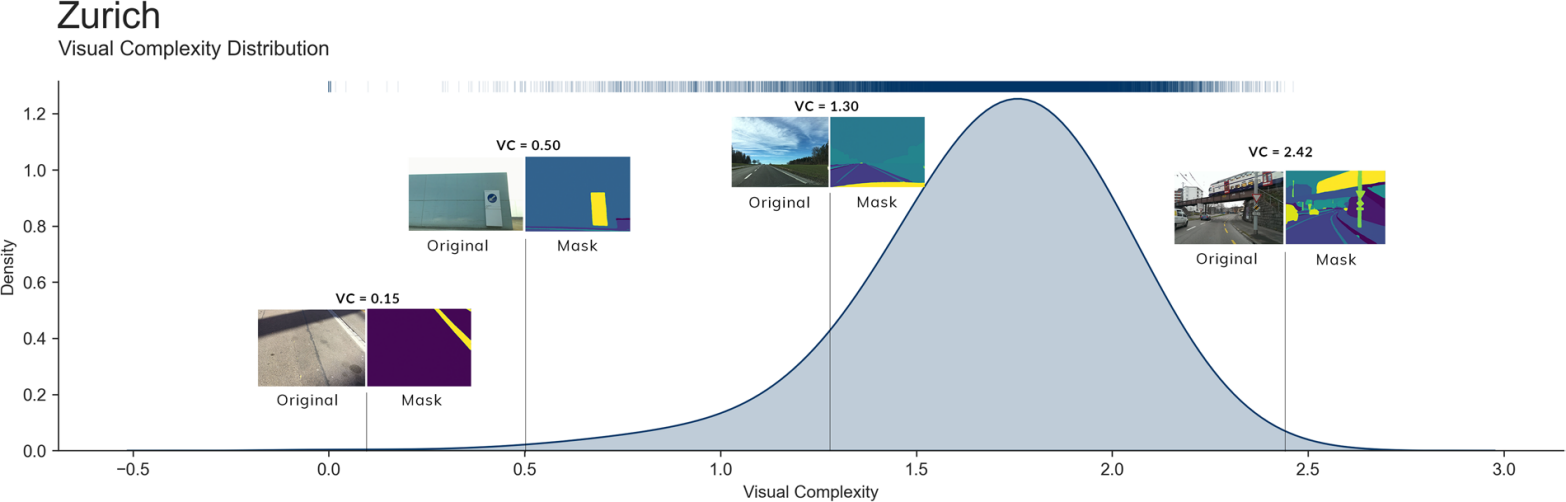
Distribution of visual complexity within streetview images in Zurich. Visual complexity corresponds to information cross entropy of semantic classes in each image. We show the visual complexity distribution for the entire image set of Zurich, Switzerland (N = 18,565). Images with low visual complexity reveal little semantic information on streetscapes and are removed. Source of imagery: Mapillary.

People contribute different types of images to crowdsourced street-level imagery, including images taken from different angles such as front-facing, side-facing, overhead, and panoramic^84^. However, segmentation models are typically trained on front-facing imagery. Therefore, other perspective views can skew segmentation results due to object appearance distortion. To manage image perspective, we first determine the orientation of all network edges and subsequently exclude images with an orientation angle deviation exceeding 20 degrees (selected following visual inspection) from their respective network edges. To ensure adequate daylight visibility, we narrow the image set to those captured between 9 am and 5 pm (local time), using image metadata. In some rare instances, we encountered images with severe occlusion, such as a street view obstructed by a large bus or street furniture. To tackle this issue, we have devised a post-segmentation heuristic that utilises visual complexity (information cross entropy on semantic classes) to identify and eliminate problematic images. This approach works intuitively by detecting images with low semantic information (e.g., where the majority of pixels correspond to a vehicle). Figure 5 displays examples of problematic images and their respective visual complexity values for Zurich, Switzerland. Based on our experiments, we determined that a cross entropy threshold of 1.0 is effective in filtering out such images.

Last but not least, where there are many images within a tile, we reduce computational workload by downsampling the available pool of images. More specifically, we apply random proportional sampling (10%) to each tile and set a minimum image count threshold of 250. In total, we segmented approximately 4 million images spanning 50 cities out of an initial selection of 97 million images. An overview of the image screening and selection process is enumerated for each city (see Table 3).

**Table 3:** SVI pre-processing process for each city.

City	Adelaide	Amsterdam	Antwerp	Athens	Atlanta	Auckland	Austin	Barcelona
**Initial**	2,802,291	286,395	5,402,430	1,537,459	625,164	353,156	2,162,429	189,797
**Excluded**								
Alignment	1,075,263	120,273	2,655,748	1,011,414	177,073	134,228	1,075,652	90,673
Daylight	888,111	49,600	1,293,082	123,986	184,432	140,102	579,929	31,642
Sampling	730,387	92,623	1,268,032	357,115	233,294	61,247	451,865	57,234
**Final Set**	108,530	23,899	185,568	44,944	30,365	17,579	54,983	10,248
City	Belo Horizonte	Berlin	Bern	Brisbane	Bogota	Boston	Brisbane	Budapest
**Initial**	1,902,895	9,182,485	93,118	3,009,352	3,379,425	997,005	263,839	4,476,229
**Excluded**								
Alignment	937,815	5,670,429	43,448	1,475,597	2,125,875	224,526	83,826	1,626,411
Daylight	342,504	965,366	14,612	892,280	408,076	265,494	118,196	809,197
Sampling	550,240	2,261,792	29,382	562,938	757,650	452,731	41,399	1,826,857
**Final Set**	72,336	284,898	5,676	78,537	87,824	54,254	20,418	213,764
City	Buenos Aires	Campinas	Chiang Mai	Chicago	Denver	Edinburgh	Glasgow	Hanoi
**Initial**	1,924,268	3,434,931	1,031,741	1,299,032	715,003	393,155	141,249	245,814
**Excluded**								
Alignment	1,313,143	1,398,166	403,326	392,735	174,572	166,563	54,996	161,716
Daylight	200,061	530,400	329,385	478,447	200,910	70,200	14,762	55,664
Sampling	332,076	1,329,840	266,639	367,300	294,617	124,503	53,872	24,882
**Final Set**	78,988	176,525	32,391	60,550	44,904	31,889	17,619	3,552
City	Houston	Johannesburg	Kowloon	Kuala Lumpur	Madrid	Melbourne	Mexico	Miami
**Initial**	1,111,777	1,861,856	121,673	1,823,540	524,564	2,872,774	5,265,189	195,989
**Excluded**								
Alignment	132,394	1,229,335	74,692	1,233,384	212,099	690,406	2,945,509	60,178
Daylight	222,493	138,620	17,325	142,977	94,249	586,015	588,918	32,637
Sampling	658,793	416,163	25,423	400,711	160,571	1,432,388	1,550,496	91,368
**Final Set**	98,097	77,738	4,233	46,468	57,645	163,965	180,266	11,806
City	Milan	Paris	Phoenix	San Jose	Santiago	Sao Paulo	Sapporo	Seattle
**Initial**	1,035,534	275,138	5,573,756	129,054	1,326,959	8,729,024	345,355	2,219,755
**Excluded**								
Alignment	370,120	128,857	1,504,118	56,746	880,883	4,038,022	91,713	767,680
Daylight	307,417	68,199	2,058,236	39,656	101,677	1,166,079	93,805	525,040
Sampling	318,062	64,471	1,792,031	27,456	291,824	3,150,399	128,706	829,213
**Final Set**	39,935	13,611	219,371	5,196	52,575	374,524	31,131	97,822
City	Singapore	Sydney	Taichung	Taipei	Tokyo	Toronto	Washington	Yokohama
**Initial**	913,358	1,891,064	428,610	1,689,293	3,610,395	1,290,948	5,623,925	1,436,846
**Excluded**								
Alignment	396,483	1,192,311	168,850	1,197,065	1,754,845	299,357	1,404,115	259,128
Daylight	99,205	433,596	91,065	276,056	402,605	221,769	1,628,221	232,547
Sampling	367,934	170,299	122,749	188,950	1,299,839	673,961	2,331,651	847,190
**Final Set**	49,736	94,858	45,946	27,222	153,106	95,861	259,938	97,981
City	Zagreb	Zurich						Total (50 cities)
**Initial**	656,242	333,735						**97,135,015**
**Excluded**								
Alignment	188,262	125,521						**43,995,541**
Daylight	155,157	60,402						**18,770,404**
Sampling	267,878	129,247						**30,266,288**
**Final Set**	44,945	18,565						**4,102,782**

### Automated checks for construct validity

To ensure construct validity at the level of individual data components, we employ a series of automated checks. For buildings, we programmatically validate each building footprint entry and use a procedural script to merge multiple polygons into a single one. For networks, we eliminate self-connections, duplicate nodes, and edges. Additionally, we verify that the network is a fully connected subgraph. For urban population data, we systematically compare aggregate figures with available census data to confirm the reliability of our estimates. To address the integration of various spatial data layers at different scales, we utilise an interactive and visually-oriented data processing pipeline to maintain spatial consistency. Each subsequent data layer undergoes visual cross-validation against its target boundary before we proceed with any spatial computation.

## Usage Notes

**Fig. 6:**
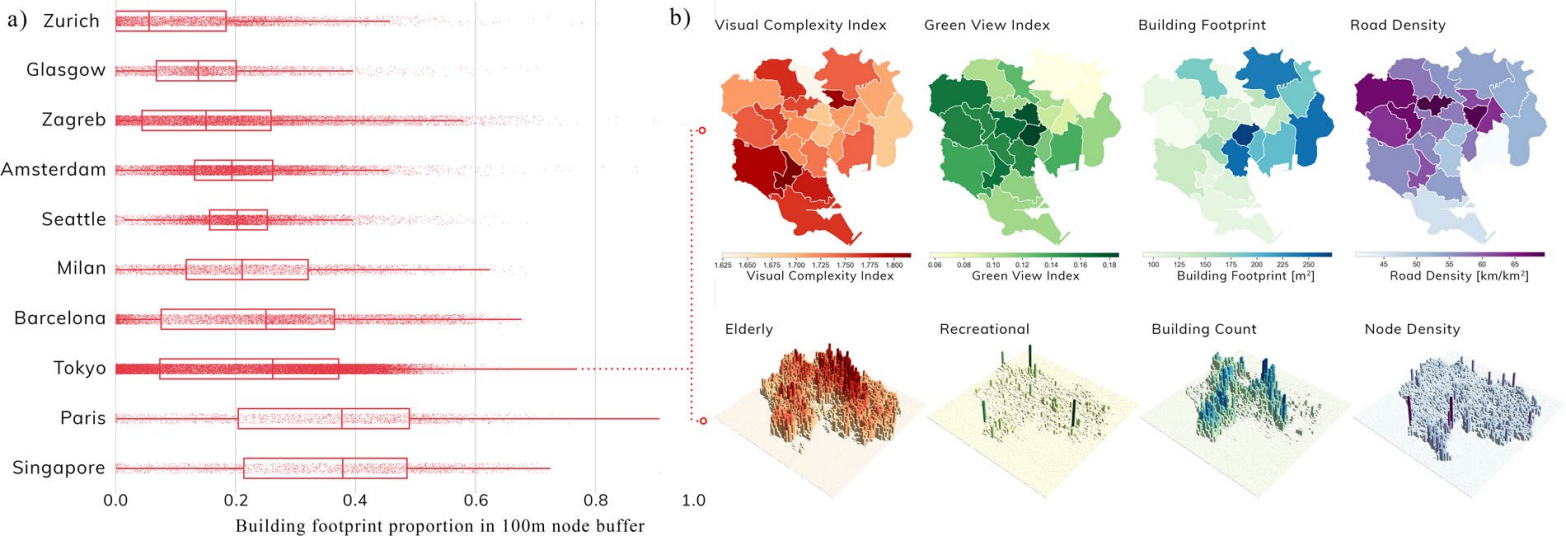
Multiscalar descriptive analysis of network indicators. Network indicators can be used for multi-scalar descriptive analyses of cities. (**a**) Comparative analysis of the distributions of building footprint proportions across network nodes with a 100-meter catchment area in ten cities. (**b**) Visualizing the spatial distribution of urban context and semantics layers at various levels of aggregation in Tokyo. Sources of the data samples: (**c**) OpenStreetMap contributors, Mapillary, and Meta.

Urbanity network data can be utilised for a wide range of descriptive and predictive urban network analytical tasks. Descriptive use cases include understanding linear associations between network indicators, multi-criteria location analysis, examining the similarity of contextual and semantic attributes across scales, and facilitating comparative analyses between various cities and their neighborhoods. Users can access many of these descriptive use cases through the Urbanity dashboard. Using Tokyo as an example, Figure 6 presents a multiscalar visualisation of various network indicators.

Network data can be incorporated into existing data science workflows with minimal pre-processing. For instance, city planners can combine population and POI indicators with local mobility data to investigate network accessibility to various urban amenities. Urban researchers can also develop better contextual and semantic understanding of networks by examining how attributes change across network structure. An example use case could be to employ network assortativity methods^85^ to evaluate distribution of urban greenery throughout the urban fabric. Network data can also be readily extended for various urban network predictive tasks. For graph machine learning use cases, users can easily transform the dataset to popular graph deep learning frameworks such as PyTorch Geometric (PyG) or Deep Graph Library (DGL). Users can extend their analysis by combining the network data with other local information, such as socio-economic indicators derived from census or local surveys. We support use case development with code notebooks (https://urbanity.readthedocs.io/en/latest/).

All source code, example notebooks, datasets, data derivatives, and technical validation code are released under open source licenses to facilitate reproducibility and use case extension. Data files are provided separately for each city and include feature sets for both network nodes and edges. Our data set is released in accessible formats to facilitate usability across different analytical environments and pipelines. Network data are released in the popular Geographic Javascript Object Notation (GeoJSON) format which can be easily loaded and visualised in various open geospatial environments such as QGIS (vector layer), R (simple features), and Python (GeoPandas). We provide example notebooks to show how users can load and visualise urban network data in our package (https://urbanity.readthedocs.io/en/latest/). Non-spatial data such as aggregated statistics for cities and subzones are provided in common comma separated values (CSV) format. Alternatively, we also provide a dashboard interface for users to explore global urban network data set.

The Urbanity network dataset^52^ is an ongoing global data effort to capture important contextual and semantic network characteristics of global cities. Till date, we have aimed to cover numerous cities across different geographical regions. Nonetheless, it is inevitable that we might have left out certain cities that are of interest. In the context of urban analytics studies, the primary concern often revolves around the availability of data. Focusing crowdsourced volunteered geographic information efforts on enhancing street view image coverage in cities across the global south would represent a significant stride towards improving overall coverage. In the meantime, subject to data availability, users can submit a request to have their city of interest included at the following discussions page (https://github.com/winstonyym/urbanity/discussions/1).

## Data Availability

Urbanity Python package source code is hosted under an open source MIT license (https://github.com/winstonyym/urbanity). Urbanity dashboard is generated with Dash version 2.7.1. with open source code (https://github.com/winstonyym/urbdash).
